# Proteomic Analysis Reveals Differentially Regulated Protein Acetylation in Human Amyotrophic Lateral Sclerosis Spinal Cord

**DOI:** 10.1371/journal.pone.0080779

**Published:** 2013-12-02

**Authors:** Dong Liu, Chaoxu Liu, Junqiang Li, Kazem Azadzoi, Yun Yang, Zhou Fei, Kefeng Dou, Neil W. Kowall, Han-Pil Choi, Fernando Vieira, Jing-Hua Yang

**Affiliations:** 1 Visiting scholars from Departments of Hepatobiliary Surgery, Gastroenterology and Neurosurgery, Xijing Hospital, Fourth Military Medical University, Xian, Shaanxi, China; 2 Departments of Surgery, Neurology and Urology, VA Boston Healthcare System and Boston University School of Medicine, Boston, Massachusetts, United States of America; 3 Cancer Research Center, Shandong University School of Medicine, Jinan, Shandong, China; 4 Proteomics Facilities, VA Boston Healthcare System and Boston University School of Medicine, Boston, Massachusetts, United States of America; 5 ALS Therapy Development Institute, Cambridge, Massachusetts, United States of America; Inserm, France

## Abstract

Amyotrophic lateral sclerosis (ALS) is a progressive fatal neurodegenerative disease that primarily affects motor neurons in the brain and spinal cord. Histone deacetylase (HDAC) inhibitors have neuroprotective effects potentially useful for the treatment of neurodegenerative diseases including ALS; however, the molecular mechanisms underlying their potential efficacy is not well understood. Here we report that protein acetylation in urea-soluble proteins is differently regulated in post-mortem ALS spinal cord. Two-dimensional electrophoresis (2-DE) analysis reveals several protein clusters with similar molecular weight but different charge status. Liquid chromatography and tandem mass spectrometry (LC-MS/MS) identifies glial fibrillary acidic protein (GFAP) as the dominant component in the protein clusters. Further analysis indicates six heavily acetylated lysine residues at positions 89, 153, 189, 218, 259 and 331 of GFAP. Immunoprecipitation followed by Western blotting confirms that the larger form of GFAP fragments are acetylated and upregulated in ALS spinal cord. Further studies demonstrate that acetylation of the proteins additional to GFAP is differently regulated, suggesting that acetylation and/or deacetylation play an important role in pathogenesis of ALS.

## Introduction

Amyotrophic lateral sclerosis (ALS) is an incurable neurodegenerative disease that typically leads to progressive paralysis and death within a few years of onset. However, the mechanism underlying the selective motor neuron degeneration of ALS has remained elusive. Several toxic mechanisms have been reported, including protein misfolding and aggregation [1]–[3], oxidative stress [4]–[6], glutamate excitotoxicity [2], [7]–[10], neuro-inflammation [11]–[13], mitochondrial dysfunction, and different environmental and/or genetic factors that lead to selective motor neuron damage [14]–[21]. These diverse toxic mechanisms may contribute to non-cell autonomous motor neuron damage [22], or toxicity by non-neuronal glial cells such as astrocytes and microglia [22]. Interestingly, toxicity incurred directly within motor neurons is a central contributor to disease initiation, but only a minor contributor to disease progression [23]. Conversely, toxicity incurred in non-neuronal neighboring cells may amplify the initial insult and drives rapid disease progression, but may not be sufficient to initiate the disease [23]–[27]. The precise cause of most ALS is still largely unknown.

A well-known hereditary factor is the genetic abnormality on chromosome 21 coding for copper-zinc superoxide dismutase (SOD1), which is associated with approximately 20% of familial cases of ALS or 2% of all ALS cases. Recent reports demonstrate mutations over a dozen of different proteins (TDP-43, TAR DNA-binding protein 43; FUS, Fused in Sarcoma; Ubiquilin-2, etc.) from ALS patients [14], [28]–[31]. The high degree of mutations found in apparently “sporadic” ALS cases without family history suggests that genetics plays a more significant role than previously speculated. Markedly, protein aggregation is found as a pathological hallmark for all ALS and a common feature for many neurodegenerative diseases such as Alzheimer and Parkinson diseases [32], [33]. Because the insoluble protein aggregate is found just before or at the same time that ALS symptoms begin, it can be at least one of the causes for diverse neurotoxic responses. The SOD1 mutation is sufficient to induce non-cell autonomous motor neuron killing by an unknown gain of toxicity [8], [24], [34]. Further studies demonstrate that the dominant SOD1 mutant is misfolded and aggregated into cytoplasmic inclusion bodies [34]–[37]. SOD1 aggregation into insoluble complexes is also an early event in the pathogenic process [25], suggesting that SOD1 aggregation contributes to the toxic responses. These observations imply that the common motor neuron toxicity in ALS may be associated with the abnormal protein aggregation or any cause that leads to accumulation of aggregates or blockage of aggregate clearance.

Notably, expression of the aggregation-prone mutant SOD1 has been recently demonstrated to promote tubulin acetylation, suggesting that HDAC6 impairment might be a common feature in various subtypes of ALS [38]. Indeed, HDAC inhibitors have been discovered as potential neuroprotective agents for the treatment of neurodegenerative disorders including ALS [39]–[42]. However, a major limitation lies in the broad spectrum of toxic side effects and even adverse effects. We hypothesize that the toxic side effects are due to the non-specificity of the HDAC inhibitors that may change the acetylation status of yet undefined substrates of deacetylases and/or products of acetylases, particularly in the insoluble protein aggregates relevant to the pathogenesis of ALS. In this study, we report protein acetylation recording in post-mortem spinal cord tissues with or without ALS using tandem mass spectrometry.

## Materials and Methods

### Ethics Statement

This study involved human post-mortem tissues requested from the VA Biorepository ALS Brain Bank (CSP501) under our institutional IRB guidelines in accordance with The Code of Ethics of the World Medical Association (Declaration of Helsinki) for experiments involving humans and Uniform Requirements for manuscripts submitted to Biomedical journals.

### Reagents and Instruments

RIPA buffer (Cell Signaling), PlusOne Urea (GE Healthcare), Trypsin (Sigma Aldrich), dialysis membrane (Spectrum, MWCO = 3,500), and Magic C18 5u 100A column (Michrom Bioresources) were purchased commercially. Liquid chromatography and tandem mass spectrometry (LC-MS/MS) were performed on a Q-STAR Elite NanoSpray mass spectrometer (AB Sciex) equipped with 2D NanoLC (Eksigent). Western blots were digitalized with Typhoon 8600 Imager (GE Healthcare). 2-DE was performed on the 2D gel systems (BioRAD); protein spots were excised with automatic EXQuest Spot Cutter (BioRAD); and 2D and Western blotting images were analysed with PDQuest gel analysis software (BioRAD).

### Spinal Cord Tissue Samples from Post-mortems

Age-matched spinal cords with ALS and non-ALS were requested from the ALS Brain Bank (Table S1). In each case, the diagnosis of ALS was confirmed by post-mortem neuropathological examination and documentation of extensive neuronal loss and gliosis typical of ALS throughout the cervical, thoracic, and lumbar levels of the spinal cord. Spinal cord tissues were homogenized in RIPA buffer (Cell Signaling) with 8 M urea (GE Healthcare), and proceeded with sonication in a W-225 sonicator (Heat Systems Ultrasonic Inc). The tissue lysates were centrifuged at 12,000 rpm for 10 minutes at 4°C, and the supernatant were collected as whole tissue lysate (urea-soluble proteins) for further analysis. Protein concentration was examined on NanoDrop 1000 Spectrophotometer (Thermo Fisher Scientific) according to the user’s manual. The soluble and insoluble protein fractions were obtained by dialysis of the urea-soluble proteins against PBS and centrifuged at 12,000 rpm for 15 min. The supernatant was collected as the soluble protein fraction for immunoprecipitation analysis; the pellet was used as the insoluble fraction for Western blotting by re-suspension in the SDS sample buffer.

### Two-dimensional Gel Electrophoresis (2-DE)

300 µg of whole tissue lysates were resuspended in Destreak rehydration solution with IPG buffer (GE Healthcare) and applied to 18-cm DryStrips (pH 3–11, GE Healthcare) with the Protean IEF system (Bio-Rad) at 50 V, 20°C overnight. Isoelectric focusing was performed with Protean IEF at 20°C according to the manufacture’s instruction. After focusing, the IPG strips were equilibrated in 6 M urea, 20% glycerol, 2% SDS, 0.05 M Tris-HCl pH 8.8, and 2% DTT for 20 min. For the second dimension, the IPG strips were applied on the top of 10% SDS-PAGE and the proteins were resolved. The 2D gels were stained with Sypro Ruby (Sigma-Aldrich), digitalized with Typhoon 8600 imager (GE Healthcare) and analysed with the PD-Quest 8.0 (BioRad) for the differences of protein expression.

### Liquid Chromatography Tandem Mass Spectrometry (LC-MS/MS)

Protein spots of interest were excised from 2-DE gels, washed with 25 mM ammonium bicarbonate, dehydrated by washing with 25 mM ammonium bicarbonate/50% acetonitrile (ACN) and 100% ACN, and dried in SpeedVac. Proteins in the gel slices were reduced with 20 mM DTT, alkylated with 40 mM iodoacetamide (IAA) and quenched with 10 mM DTT, followed with trypsin digestion using sequence-grade modified porcine trypsin (Sigma-Aldrich) at 37°C overnight. Peptides recovered from in-gel-trypsin digestion were desalted and concentrated by C18 ZipTip (Millipore) and subjected to LC-MS/MS analysis on Q-STAR Elite mass spectrometer. The conditions for the reversed phase liquid chromatography were: 10-cm×75 micron Magic C18 column (5 micron, 100 Å, Michrom Bioresources); mobile phase A: 2% acetonitrile +98% water +0.1% formic acid; mobile phase B: 98% acetonitrile +2% water +0.1% formic acid; and the gradient, 5–35% B over 60 min, and the flow rate, 0.95 ul/min. The conditions for mass spectrometer were: survey scan 1 sec.; information dependent product ion scan, top 5 ions; exclusion time, 10 sec.; acquiring time 80 min. The MS/MS data were analysed with Analyst QS 2.0 and ProteinPilot 2.0 (AB Sciex) and search against the human protein database (Swiss-Prot.2007.04.19, 264,492 entries) with the following options: enzyme, trypsin; missed cleavage, 1; fixed modification, carbamidomethylation of cysteine; mass tolerance for precursor ions, 20 ppm; mass tolerance for fragmented ions, 0.6 Da. Variable modifications considered: acetylation at lysine, phosphorylation at serine, threonine and tyrosine. Threshold for peptide score and E-value for accepting individual MS/MS spectra were 15 and 0.01, respectively. All modification site assignments were determined by manual spectrum interpretation.

### Western Blotting and 2D Western

For regular Western blotting, whole tissue lysates or the soluble/insoluble fractions were resolved by 10–12% SDS-PAGE. The proteins in the gels were transferred to polyvinylidene difluoride (PVDF) membranes using the Semidry transfer system (BioRad). The membranes were detected with the first antibody, such as the anti-GFAP mouse monoclonal antibody (GA5, Cell signalling), the anti-acetyl-lysine rabbit polyclonal antibody (Cell Signaling), and the antibody against β-actin (Santa Cruz). The secondary antibodies such as DyLight™ 649 conjugated goat anti-mouse IgG (Thermo Scientific) and DyLight™ 649 conjugated goat anti-rabbit IgG (Thermo Scientific) were used to visualize the detected proteins. The blots were scanned with Typhoon 8600 imager (GE Healthcare) and digitalized with ImageQuant TL software (GE Healthcare). For 2D Western blotting, the spinal cord tissues were homogenized in Destreak rehydration solution with IPG buffer (GE Healthcare) by sonication with W-225 sonicator. The supernatant was collected by centrifugation at 12,000 rpm for 10 minutes at 4°C. 50 µg proteins were applied to 7-cm DryStrips (pH 4–7, GE Healthcare) with the Protean IEF system at 50 V, 20°C overnight. Isoelectric focusing of the proteins was performed with Protean IEF under the following conditions: 150 V for 0.5 h, 150–2000 V for 1 h, and 2000 V for 4 h. The second dimension was performed with a mini-gel system (BioRad). The proteins on the gels were transferred to PVDF membranes and analysed by Western blotting as previously described.

### Immunoprecipitation (IP) and IP-Western

For immunoprecipitation, the soluble protein fractions were prepared from the whole tissue lysates by dialysis against PBS and centrifugation. Alternatively, for IP-Western, the soluble proteins were prepared by homogenization of the spinal cord tissues in the RIPA buffer (GE Healthcare) without urea. The supernatant was collected by centrifugation at 12,000 rpm for 10 minutes at 4°C. 100 µg of the soluble proteins from each group were incubated with the anti-GFAP antibody or the antibody against acetyl-lysine (Cell Signaling). The proteins associated with the antibodies were precipitated with Protein G Sepharose (GE Healthcare) according to the user’s manual. After washing, the immunoprecipitated proteins were resolved by SDS-PAGE and stained with Sypro Ruby (Sigma-Aldrich). For IP-Western, the immunoprecipitated proteins were resolved by SDS-PAGE, transferred to PVDF membrane and detected with the anti-GFAP antibody or the antibody against acetyl-lysine as previously described.

## Results

### Differentially Expressed Protein Clusters in ALS and Non-ALS Spinal Cords

**Figure 1:**
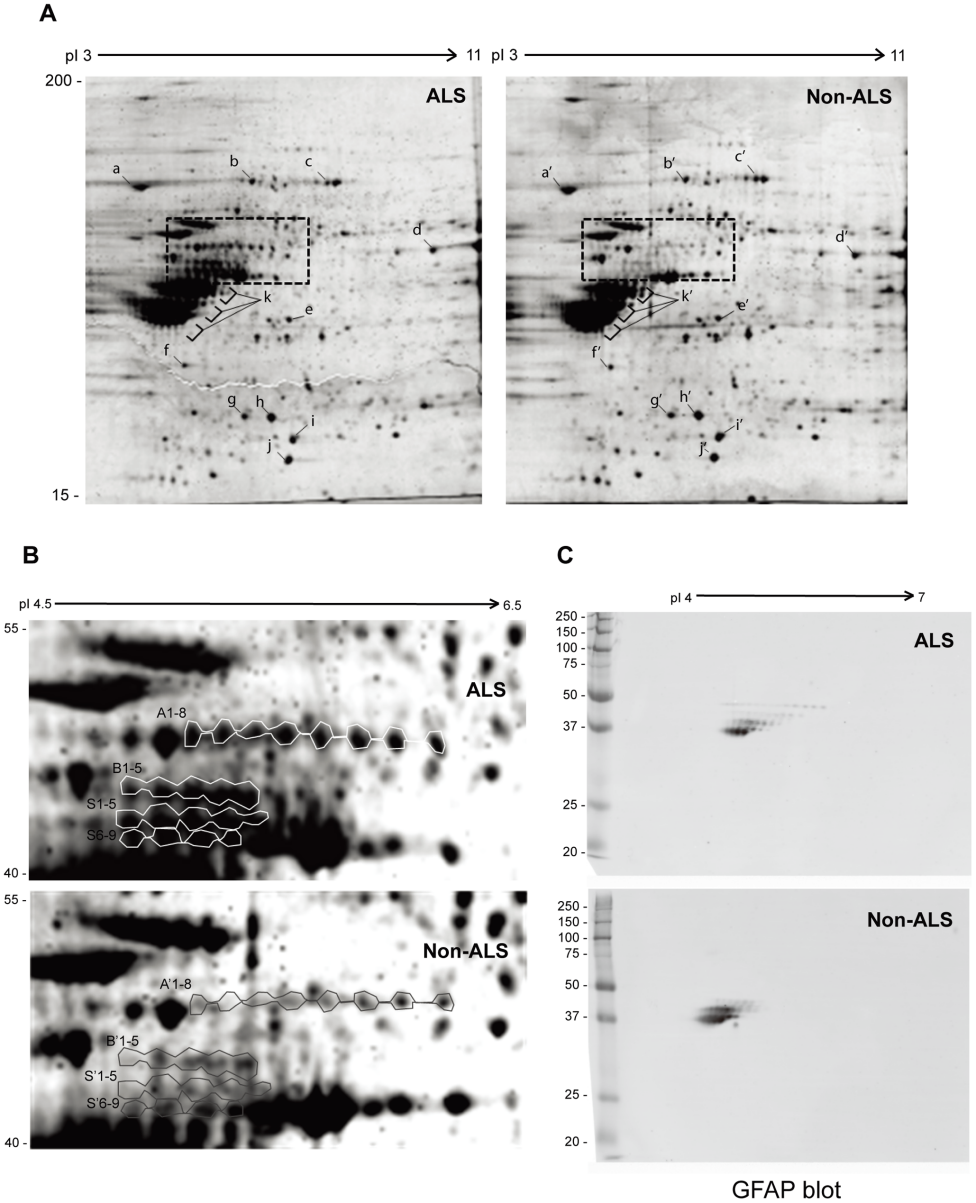
Comparison of urea-soluble proteins from ALS and non-ALS spinal cords by 2D SDS-PAGE. (**A**) Urea-soluble whole tissue lysates were prepared from pooled ALS or non-ALS spinal cords in the RIPA lysis buffer containing 8 M of urea. The first dimension was 18-cm immobilized pH gradient isoelectric focusing (IEF) from pI = 3–11; the second dimension was 10% SDS-PAGE. The gels were stained with Sypro Rubby. High quality spots marked with a–i and a’–i’ were randomly selected as references to normalize the differences between different gels. (**B**) Differentially expressed protein clusters between ALS and non-ALS spinal cords. The cluster A, B and S from ALS and A’, B’ and S’ from non-ALS were excised from the 2-D gels and subjected to LC-MS/MS protein identification. (**C**) Western blotting analysis of the protein clusters with anti-GFAP antibody. The urea-soluble whole tissue lysates were resolved by mini-2D SDS-PAGE, transferred to the PVDF membrane and detected with the antibody against GFAP.

Age-matched post-mortem spinal cords with ALS (n = 4) and non-ALS (n = 4) were requested from ALS Brain Bank. Whole tissue lysates were prepared in RIPA buffer containing high concentration of urea to increase the solubility of the protein aggregates. 300 µg of pooled urea-soluble proteins (whole tissue lysates) were analysed by 2D SDS-PAGE as described in Materials and Methods. No significant change was observed for most proteins; however, a few laddered protein clusters with molecular weight between 40–55 kDa, indicated as A1-8, B1-5, S1-5 and S6-9 (**Fig. 1**), were significantly different in ALS and non-ALS. Notably, the proteins in each ladder were apparently similar in molecular weight, but different in the isoelectric points, suggesting different modifications on the charged residues in association with ALS. To identify these proteins, gel slices from the clusters were excised and digested with trypsin; peptides were analyzed by LC-MS/MS. The results are summarized in **Table 1**. Interestingly, glial fibrillary acidic protein (GFAP) was dominant in all clusters (**Fig. S1**), while a few other neurofilament proteins such as NFL and Vimentin were also identified (**Fig. S2, S3**). Because each ladder of spots had 4–8 proteins apparently similar in molecular weight but different in isoelectric points, we reasoned that different ladders were from truncated/degraded GFAP fragments whereas the spots with similar molecular weight were due to varying degrees of modification at the charged residues.

**Table 1:** Identification of Protein Spots on the 2D gel by LC-MS/MS^a^.

Spot #	Protein Name	MW/pI	Coverage (%)^b^	Score (MinimumScore)^c^
A1-8	GFAP: Glial fibrillary acidic protein	50/5.4	38	302 (32)
	VIME: Vimentin	54/5.1	10	50 (32)
B1-6	GFAP: Glial fibrillary acidic protein	60/4.1	20	538 (31)
	NFL: Neurofilament light polypeptide	61/4.6	5	113 (31)
	VIME: Vimentin	54/5.1	30	43 (31)
S1-9	GFAP: Glial fibrillary acidic protein	60/4.1	10	538 (36)
	NFL: Neurofilament light polypeptide	61/4.6	5	113 (36)

a The differentially expressed protein spots on the 2-D gels of ALS were excised and proteins identified by LC-MS/MS.

b The length of identified peptide fragments divided by the length of the protein.

c Mascot algorithm score; the minimum score is required to have a statistic significance (p<0.05).

To confirm the laddered protein clusters were from GFAP, the urea-soluble proteins were resolved by mini-2D SDS-PAGE, transferred on PVDF membrane and detected with the antibody against GFAP (**Fig. 1C**). Indeed, the laddered GFAP clusters were detected. Particularly, the larger forms of GFAP fragments were clearly found in ALS spinal cord, whereas the small forms were slightly more in non-ALS samples.

### Heavily Acetylated GFAP in ALS Spinal Cord

**Figure 2:**
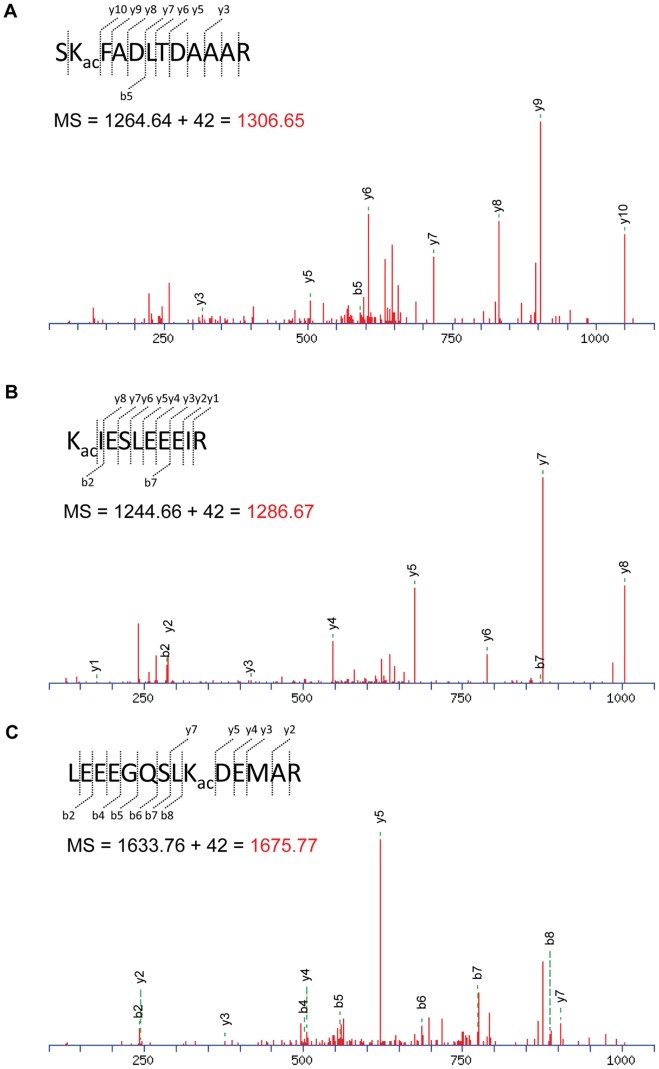
Identification of lysine acetylation in GFAP. The LC-MS/MS data from the protein clusters potentially with different charges were subjected to phosphorylation and acetylation modification analysis. Lysine acetylation was identified on several GFAP peptides. High-resolution MS/MS spectra of three tryptic peptides SKFADLTDAAAR (**A**), KIESLEEEIR (**B**), and LEEEGQSLKDEMAR (**C**) are shown. K_ac_ indicates the acetylated lysine residue. The identified peptide with a mass shift of 42 Da is shown on the top of the MS/MS spectra.

**Figure 3:**
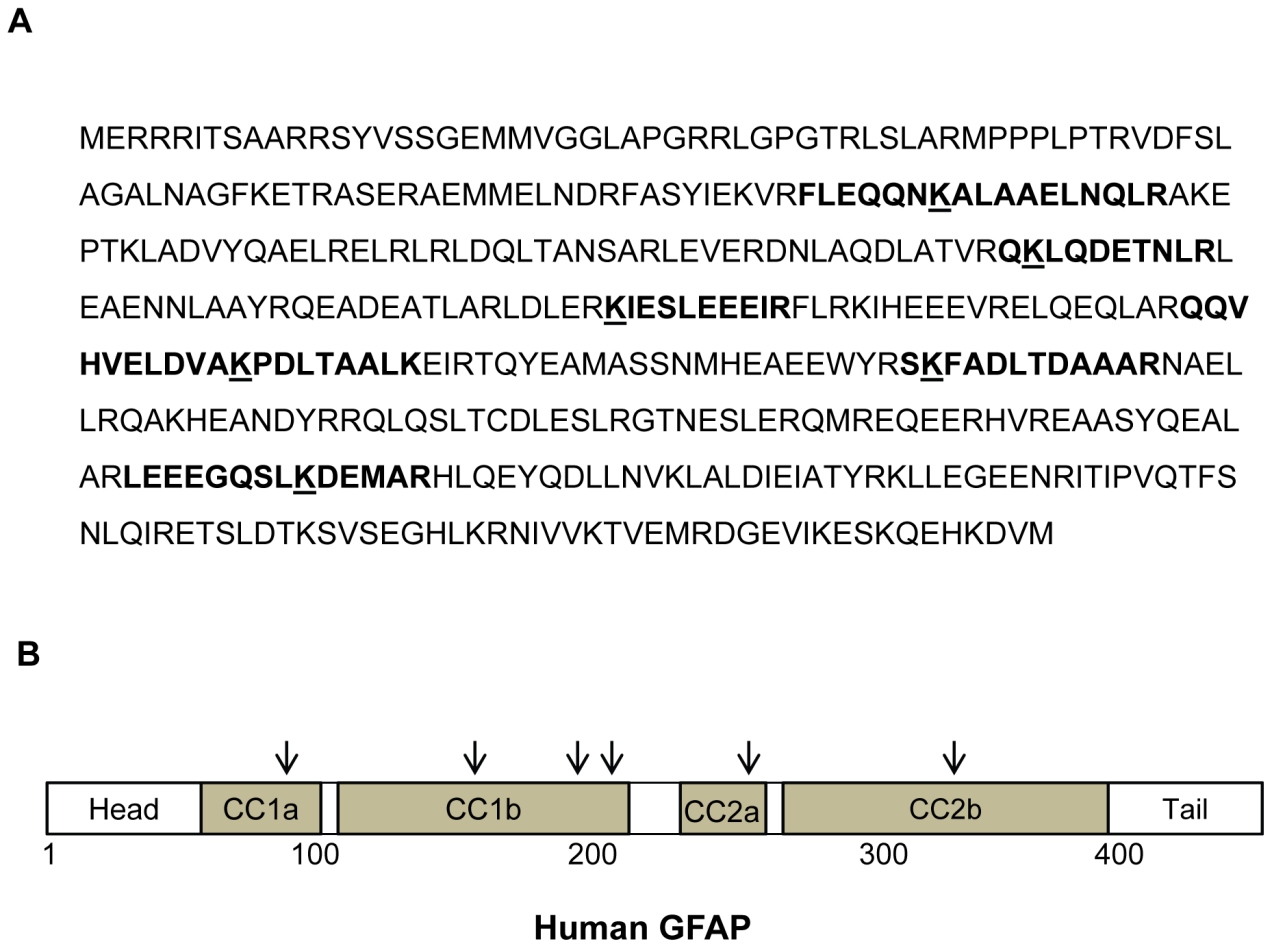
The acetylated lysine residues in GFAP. (**A**) The GFAP sequence with six identified positions for lysine acetylation. Bold sequence, the identified tryptic peptide; underlined K, the acetylated lysine residue. (**B**) A schematic diagram of human GFAP structure with four α-helical coiled-coil domains (CC1a, CC1b, CC2a, CC2b) and the positions for acetylation (arrows). The acetylated lysines fell into the highly conserved coiled-coil domains.

Because the laddered GFAP appeared as a few clusters of differently charged fragments, we hypothesized that these GFAP fragments were probably modified at the hydroxyls of serine/threonine/tyrosine residues by phosphorylation, and/or the amino group of lysine residue by acetylation. To test this hypothesis, the urea-soluble proteins were resolved and the dominant GFAP bands were excised and digested with trypsin. The tryptic GFAP peptides were analyzed by LC-MS/MS with the option of modification for acetylation and phosphorylation. GFAP was identified with 73% coverage (**Fig. S1**). Although no phosphorylation was observed within this sequence range, six peptides containing the acetylated lysine residue were identified (**Table 2** **)**. MS/MS spectra of three identified peptides with acetylated lysine were shown in **Fig. 2**. Acetylated lysines were spread within the α-helical coiled-coil domain of the filament structure [43] at positions 89, 153, 189, 218, 259 and 331 (**Fig. 3**). Considering that a few acetylated lysines might have been missed in the uncovered GFAP sequence, the six acetylated lysine positions fairly matched the pattern of the laddered GFAP clusters, i.e. 4–8 protein spots of the similar molecular weight on the 2D gels (**Fig. 1**). Thus, it supported our hypothesis that the truncated GFAP fragments in each ladder might have different degrees of acetylation at the identified lysine sites.

**Table 2:** Acetylated Lysine and Peptides of GFAP Identified by LC-MS/MS^a^.

Acetylated Peptide^b^	Neutral MS^c^		
Position: Sequence	Calculated	Observed	Mascot Score^d^
259: SKFADLTDAAAR	1264.64	1306.65	58
189: KIESLEEEIR	1244.66	1286.67	57
153: QKLQDETNLR	1243.65	1285.66	25
331: LEEEGQSLKDEMAR	1633.76	1675.77	15
89: FLEQQNKALAAELNQLR	1985.07	2027.08	47
218: QQVHVELDVAKPDLTAALK	2074.14	2116.15	18

a Acetylation was confirmed by MS and MS/MS of the peptide.

b Numbering according Genbank accession # P14136; underline indicates the acetylated lysine.

c Monoisotopic mass of the neutral peptide.

d Mascot algorithm score of each acetylated peptide.

### Differentially Distributed Larger GFAP Fragments between the Soluble and Insoluble Protein Fractions of ALS and Non-ALS Spinal Cords

**Figure 4:**
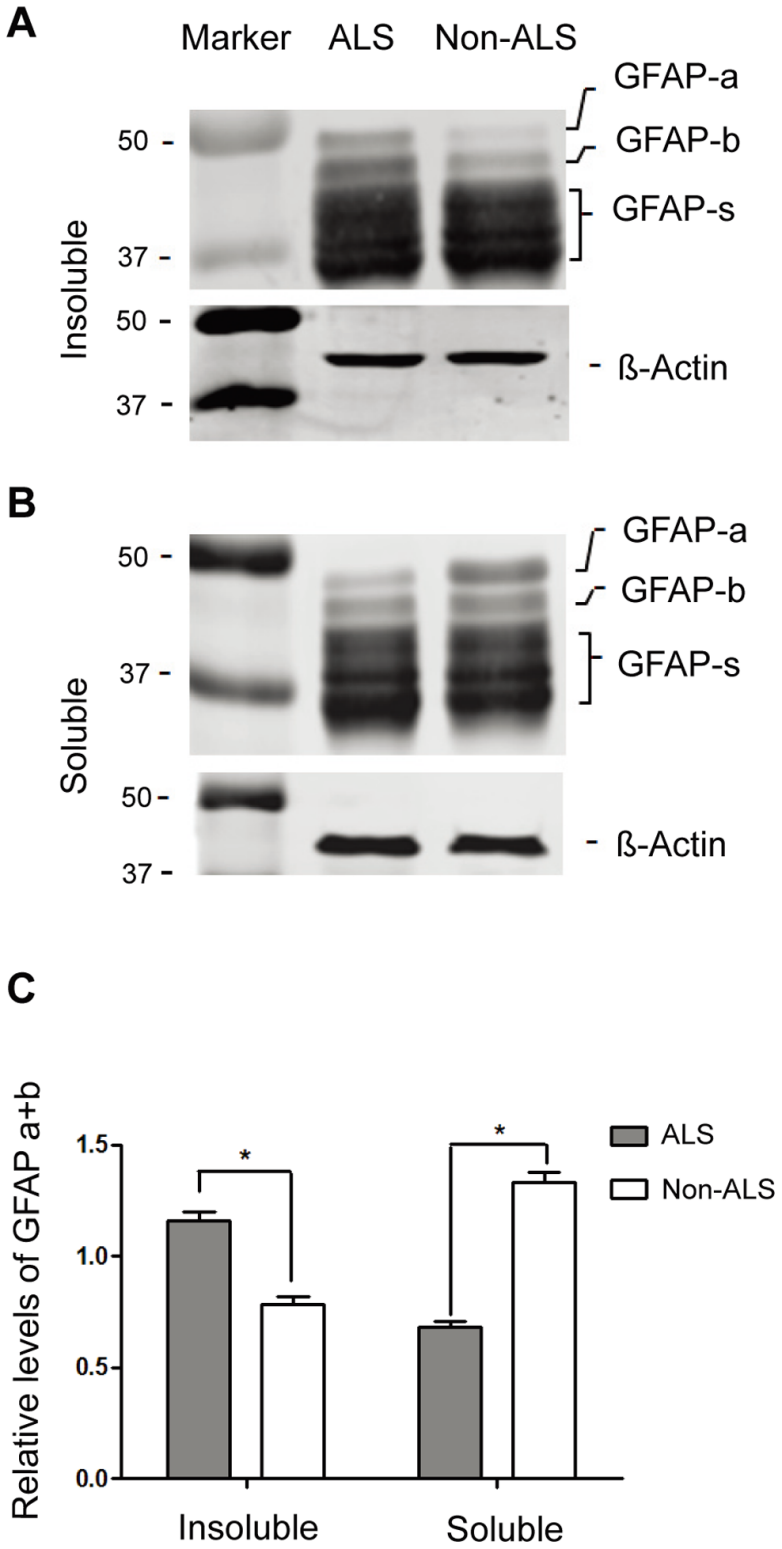
Western Blotting analysis of the GFAP fragments in the soluble and insoluble protein fractions of ALS and non-ALS spinal cords. (**A, B**) Western Blotting analysis of the GFAP fragments in the insoluble (A) and soluble (B) fractions. The urea-soluble proteins were dialyzed against PBS and centrifuged. The pellet (insoluble fraction) and the supernatant (soluble fraction) were analyzed by Western blotting and detected with the anti-GFAP antibody. GFAP-a and GFAP-b, the larger forms of GFAP; GFAP-s, the degraded GFAP fragments; β-actin, internal control. (**C**) Quantitation of two larger forms of GFAP fragments. The expression levels of GFAP-a and GFAP-b relative to β-actin were calculated. The large forms of GFAP are preferably found in the insoluble fractions (* p<0.05, n = 4).

The most apparent effect of lysine acetylation is the inhibition of proteasome-mediated protein degradation [44]–[46], and accumulation of insoluble protein aggregate in the motor neuron and astrocytes was a hallmark commonly associated with ALS [47], [48]. Thus, we hypothesized that lysine acetylation found in ALS spinal cords might be involved in inhibition of aggregate degradation. The urea-soluble proteins were divided to soluble and insoluble fractions as described in Material and Methods and analysed separately by SDS-PAGE and immune blotting using the anti-GFAP antibody. While different GFAP fragments were consistently observed, the larger forms of GFAP fragments were differently distributed between the soluble and insoluble protein fractions in ALS and non-ALS spinal cords (**Fig. 4A, B**). In the insoluble fraction, the larger forms of GFAP (GFAP-a, b) were found more in ALS than in non-ALS spinal cord (**Fig. 4C**). In the soluble fraction, however, they were found more in non-ALS than ALS. These findings were supportive of our hypothesis that, in ALS, the larger forms of GFAP were probably more resistant to the degradation process and thus accumulated in the insoluble protein aggregate.

### Enhanced Acetylation of the Larger GFAP Fragments in ALS Spinal Cord

**Figure 5:**
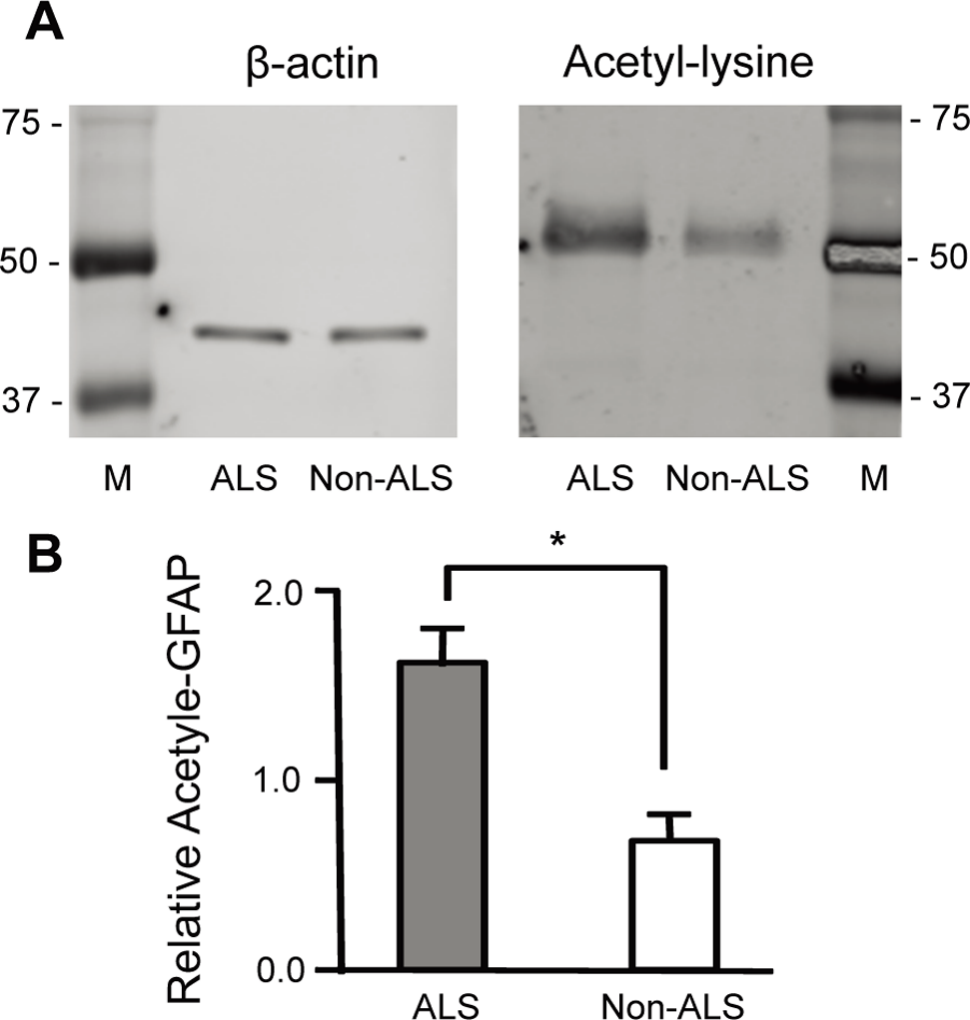
Immunoprecipitation followed by Western blotting confirms the up-regulation of the acetylated GFAP in ALS. The GFAP fragments in the soluble fractions from three individual ALS and non-ALS spinal cord samples were immunoprecipitated with the anti-GFAP antibody, and analysed by Western blotting using the antibody against acetyl-lysine (the right panel). Western blotting with β-actin was used as a control for the inputted proteins (the left panel). (**B**) Quantification of the acetylated GFAP. The relative acetylated GFAP to β-actin were compared between each pair of ALS and non-ALS samples (*ALS/non-ALS = 2.1±0.2, p<0.003, n = 3).

We further confirmed whether the immunoprecipitated GFAP fragments were acetylated by IP-Western (immunoprecipitation followed by Western blotting) using the antibodies against GFAP and acetyl-lysine, respectively. Because the insoluble proteins were not applicable for immunoprecipitation, only the soluble fractions were used. As shown in **Fig. 5**, although several GFAP fragments were immunoprecipitated, not all fragments were acetylated. The larger form(s) of GFAP fragments were dominantly detected with the acetyl-lysine antibody. Importantly, the acetylated GFAP fragments were up-regulated in the ALS spinal cord (**Fig. 5A**, the right panel, **Fig. 5B**), suggesting that deaceylation of the larger forms of GFAP were inhibited, and/or acetylation of them were enhanced in ALS spinal cord.

### Differentially Regulated Protein Acetylation in ALS and Non-ALS Spinal Cords

**Figure 6:**
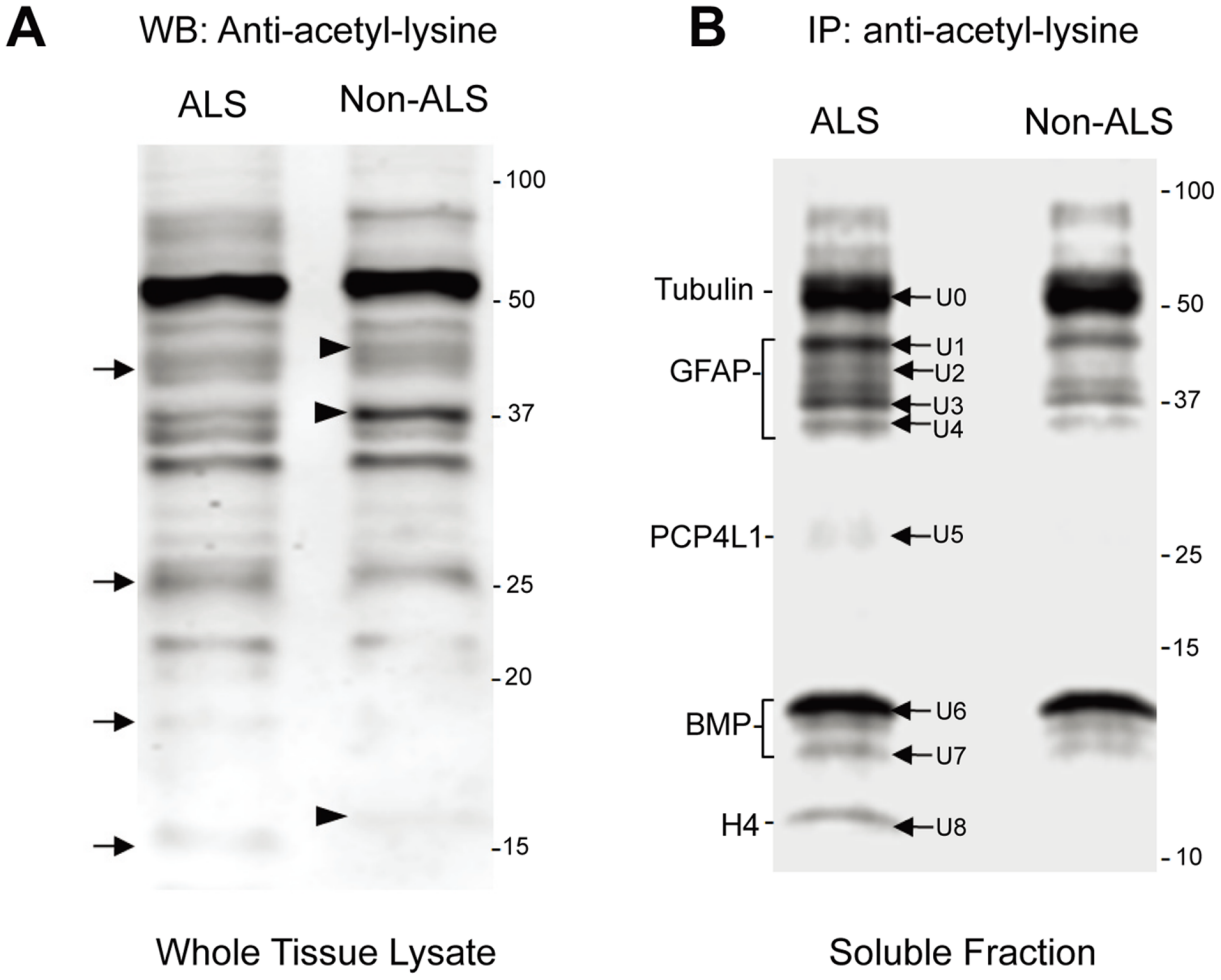
Differentially regulated protein acetylation in ALS and non-ALS spinal cords by Western blotting and immunoprecipitation. (**A**) Western blotting analysis of total acetylated proteins. The urea-soluble proteins from ALS and non-ALS spinal cords were resolved on SDS-PAGE and followed by Western blotting using the antibody against acetyl-lysine. Arrows indicate bands found in ALS spinal cords, while arrowheads indicate the bands found in non-ALS counterparts. (**B**) Immunoprecipitation of the acetylated proteins. The soluble protein fractions were immunoprecipitated with the antibody against acetyl-lysine, resolved by SDS-PAGE and stained with Sypro Ruby. The protein bands labelled with U0, U1, … U8 were recovered, digested with trypsin and identified with LC-MS/MS. The proteins that were identified by LC-MS/MS are indicated to the left.

To compare the difference of lysine acetylation additional to GFAP, the urea-soluble proteins from ALS and non-ALS were resolved by SDS-PAGE, transferred to PVDF membrane and detected by the anti-acetyl lysine antibody. As shown in **Fig. 6A**, the patterns of the acetylated proteins in ALS and non-ALS spinal cords were different. The acetylated lanes indicated with arrows were apparently present in ALS but absent in non-ALS spinal cords. Conceivably, these acetylated proteins were either the substrates of yet undefined deacetylase (HDAC) or the products of yet undefined acetyltransferase (HAT, Histone acetyltransferases). Therefore, it was conceivable that acetylation of these proteins was activated or deacetylation of these proteins was blocked in ALS spinal cords. Markedly, there were also lanes present in non-ALS but absent in ALS spinal cords (indicated with arrowheads). This could be explained as that deacetylation of these proteins was activated or acetylation was blocked in ALS spinal cords. In order to identify the different acetylated proteins in ALS postmortem spinal cord, we immunoprecipitated possible acetylated proteins from ALS and non-ALS spinal cords using immobilized anti-acetyl lysine antibody. The immunoprecipitated proteins, theoretically the acetylated proteins, were resolved by SDS-PAGE and stained with Sypro Ruby as shown in **Fig. 6B**. In order to perform immunoprecipitation, the total urea soluble proteins were dialyzed to remove urea and the insoluble proteins; thus, the result reflected the acetylated proteins in the soluble fraction. Nevertheless, significantly more acetylated proteins were obtained from ALS spinal cord, especially in the range that was later identified as GFAP. It was consistent with the 2D gel analysis that different GFAP clusters were observed in ALS spinal cord (**Fig. 1** **)**.

### Identification of Differentially Acetylated Proteins in ALS and Non-ALS Spinal Cords

The differentially acetylated protein bands in ALS spinal cord were cut from the gel and processed with trypsin digestion followed by LC-MS/MS (**Fig. 6B**, arrows). The identified proteins were summarized in **Table 3**. U0 was found to be tubulin beta-2A (**Fig. S4**), which was well known to be heavily acetylated [49]. Detection of the acetylated tubulin was considered as an internal control for a successful immunoprecipitation of the acetylated proteins. U1, U2, U3 and U4 were the differently degraded GFAP products (**Fig. S5**), and were consistent with the previous data. This provided additional support that acetylation was involved in the regulation of GFAP degradation. Interestingly, GFAP knockout mice undergo multiple degenerative processes including abnormal myelination and disruption of blood-brain barrier [50]. U5 was PCP4L1 (**Fig. S6**), the Purkinje cell protein-4 like 1; but the difference in expression as determined by the Mascot score was not significant. U6 was identified as myelin basic protein (MBP) (**Fig. S7**), an important protein in CNS myelination. Acetylation of MBP at the N-terminal end was known; however, acetylation at its side chain has not yet been reported. U7 was identified as histone 4 (H4) (**Fig. S8**). Although HDACs and HATs were originally found in acetylation of histones, it needs further investigation to address why the acetylated H4 is enhanced in post-mortem spinal cord with ALS.

**Table 3:** Identification of the Acetylated Proteins in ALS Spinal Cord by Immunoprecipitation and LC-MS/MS^a^.

Spot #	Protein Symble	Protein Name	MW/pI	Coverage (%)	Mascot Score
U0	TBB	Tubulin beta chain	50/4.8	4	27
U1	GFAP	Glial fibrillary acidic protein	50/5.4	32	147
U2	GFAP	Glial fibrillary acidic protein	50/5.4	50	207
U3	GFAP	Glial fibrillary acidic protein	50/5.4	37	149
U4	GFAP	Glial fibrillary acidic protein	50/5.4	44	211
U5	PCP4L1	Purkinje cell protein 4 like 1	7/6.3	7	17
U6	MBP	Myelin basic protein	33/9.8	38	60
U8	H4	Histone H4	11/11.3	52	30

a The proteins that were immunoprecipitated with the antibody against acetylated lysine were resolved on SDS-PAGE and stained with Rubby-RED. The protein bands corresponding to the Western blots using the same antibody were excised and identified LC-MS/MS.

## Discussion

Our proteomic approach reveals a significant difference in protein acetylation at the lysine residues between the post-mortem ALS and non-ALS spinal cords. Because the acetylated proteins are the substrates of deacetylase (HDAC) and/or the products of acetyltransferase (HAT), our results demonstrate that yet undefined HDAC, HAT or the factors that affect their activities are impaired in ALS. Our findings are consistent with a recent study from another group reporting that ALS-linked mutant SOD1 can modulate HDAC6 activity [38], suggesting that HDAC6 impairment might be a common feature in various subtypes of ALS.

Generally, because the deacetylation activity of yet undefined HDACs specific for these proteins may be inhibited in ALS spinal cord, one may assume that inhibition of the HDAC activity, *i.e.* HDAC inhibitors, would have a toxic effect, whereas enhancement of this activity would be protective against ALS. Indeed, some HDACs have neuroprotective, neurotrophic and anti-inflammatory properties across multiple neurodegenerative diseases [42], [51]–[55]. For instance, HDAC6 has shown protective effects and certain HDAC inhibitors are shown to cause a variety of toxic side effects [51], [56]. SIRT1 deacetylase protects against neurodegeneration in models of Alzheimer’s disease as well as ALS [57], although enhancing SIRT1 activity by resveratrol did not affect functional improvement or increased longevity in an SOD1 mutant mouse model of ALS [58].

The most apparent effect of protein acetylation is the inhibition of proteasome-mediated protein degradation [44]–[46]. It is thus conceivable that deacetylation of certain acetylated proteins would ameliorate disease progression. In our study, 2-dimensional gel analysis reveals differently modified GFAP clusters of urea-soluble proteins in ALS spinal cord (**Fig. 1**). Significantly higher levels of the larger GFAP fragments are found in the insoluble fraction (**Fig. 4**), supporting the notion that protein degradation may be impaired in ALS. In consistent, immunoprecipitation followed by Western blotting with the acetyl-lysine antibody confirms that the larger forms of GFAP fragments in the soluble fractions are acetylated (**Fig. 5**). In particular, the acetylated larger GFAP fragments are upregulated in ALS spinal cord, suggesting that protein acetylation is involved in GFAP degradation in ALS.

The biological function of non-histone substrates of HDAC/HAT might have been underestimated. A question is raised whether HDAC is required for protein degradation especially under ALS condition. It has been demonstrated that genetic mutations play an important role and aggregate-prone mutations could trigger insoluble protein aggregate in ALS [38], [59]. When the production of misfolded proteins exceeds the capacity of the chaperone refolding system (CRS) and the ubiquitin-proteasome system (UPS), misfolded proteins are actively transported to cytoplasmic aggregate inclusions that will be eventually cleared by autophagy [33], [60]. Recent studies indicate that UPS targets misfolded proteins for degradation, while autophagy acts as a compensatory degradation system when UPS is blocked [59]. Interestingly, autophagy compensates for UPS dysfunction in an HDAC6-dependent manner, while HDAC6 is a microtubule-associated histone deacetylase that interacts with poly-ubiquitinated proteins and the dynein motor protein. Overexpression of HDAC6 in a fly model of neurodegenerative disease indeed accelerates the degradation of the aggregate-prone protein by autophagy and protects the flies from neurotoxicity [59]. Further investigation is needed to elucidate the spectrum of HDAC substrates of whether and how they are involved in protein degradation of the insoluble protein aggregate in ALS spinal cord.

Notably, GFAP is not previously known as a substrate of acetylation and this is the first report that GFAP is heavily acetylated. GFAP is an essential component of filament in astrocytes and plays an important role in astrocyte-neuron interactions as well as cell-cell communication. Many studies have shown that ALS is at least partially a non-cell autonomous disease and that non-motor neuron cells such as astrocytes expressing mutant hSOD1 contribute to the pathogenesis of ALS. Mutant SOD1 within non-neuronal cells including astrocytes is an important contributor to motor neuronal toxicity and disease progression [24]. Thus, because mutant SOD1 has been shown to modulate HDAC6 activity and increases tubulin acetylation [38], it is possible that GFAP acetylation is also a consequence of abnormal HDAC6 activity in astrocytes of ALS spinal cord.

## Conclusions

Protein acetylation at lysine residues is differentially regulated in ALS and non-ALS spinal cord. Because the acetylated proteins are the substrates of deacetylase and/or the products of acetyltransferase, our results demonstrate that yet undefined HDAC, HAT or the factors that affect HDAC/HAT activities are impaired in ALS spinal cord. These findings warrant further investigation to identify the responsive HAT/HDAC for possible therapeutic application.

## Supporting Information

Figure S1 **Typical MS/MS result of GFAP in spots cut from 2D gel.** Sequence coverage and matched peptides are shown in Bold. Two representativeMS/MS spectra of identified peptides were shown.
(TIF)

Figure S2 **Typical MS/MS result of Vimentin in spots cut from 2D gel.** Sequence coverage and matched peptides are shown in Bold. Two representativeMS/MS spectra of identified peptides were shown.
(TIF)

Figure S3 **Typical MS/MS result of NFL in spots cut from 2D gel.** Sequence coverage and matched peptides are shown in Bold. Two representativeMS/MS spectra of identified peptides were shown.
(TIF)

Figure S4 **Typical MS/MS result of Tubulin beta Chain in bands cut from IP-SDS-PAGE gel.** Sequence coverage and matched peptides are shown in Bold. Two representative MS/MS spectra of identified peptides were shown.
(TIF)

Figure S5 **Typical MS/MS result of GFAP in bands cut from IP-SDS-PAGE gel.** Sequence coverage and matched peptides are shown in Bold. Two representative MS/MS spectra of identified peptides were shown.
(TIF)

Figure S6 **Typical MS/MS result of PCP4L1 in bands cut from IP-SDS-PAGE gel.** Sequence coverage and matched peptides are shown in Bold. Two representative MS/MS spectra of identified peptides were shown.
(TIF)

Figure S7 **Typical MS/MS result of Myelin basic protein in bands cut from IP-SDS-PAGE gel.** Sequence coverage and matched peptides are shown in Bold. Two representative MS/MS spectra of identified peptides were shown.
(TIF)

Figure S8 **Typical MS/MS result of Histone H4 in bands cut from IP-SDS-PAGE gel.** Sequence coverage and matched peptides are shown in Bold. Two representative MS/MS spectra of identified peptides were shown.
(TIF)

Table S1 **Post-mortem spinal cord tissues used in the present study.** Age-matched postmortem spinal cords were dissected within 48 hours. ∼5 mm^3^ of the same tissues were prepared as described in Materials and Methods. Aliquots were stored at −80°C until use.
(DOC)
