# Supplementary figures and images for: Proteomic Analysis Reveals Differentially Regulated Protein Acetylation in Human Amyotrophic Lateral Sclerosis Spinal Cord
Source: PLoS One. 2013 Dec 2;8(12):e80779. doi: 10.1371/journal.pone.0080779 (PMC3846615; doi:10.1371/journal.pone.0080779)

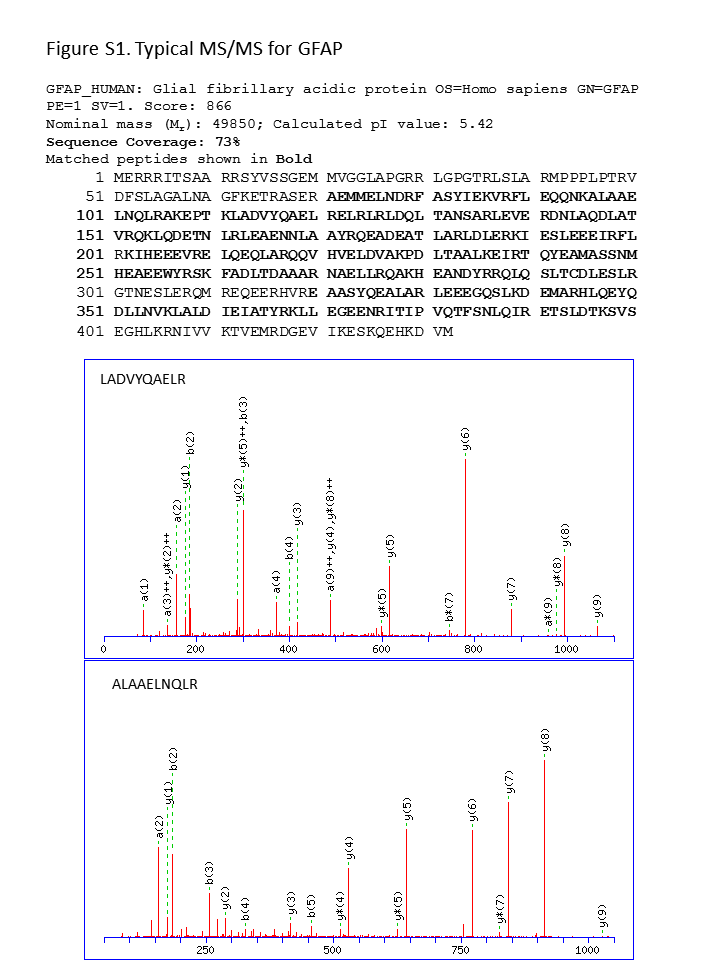

Supplement: Figure S1 — Typical MS/MS result of GFAP in spots cut from 2D gel. Sequence coverage and matched peptides are shown in Bold. Two representativeMS/MS spectra of identified peptides were shown. (TIF) [file pone.0080779.s001.tif]

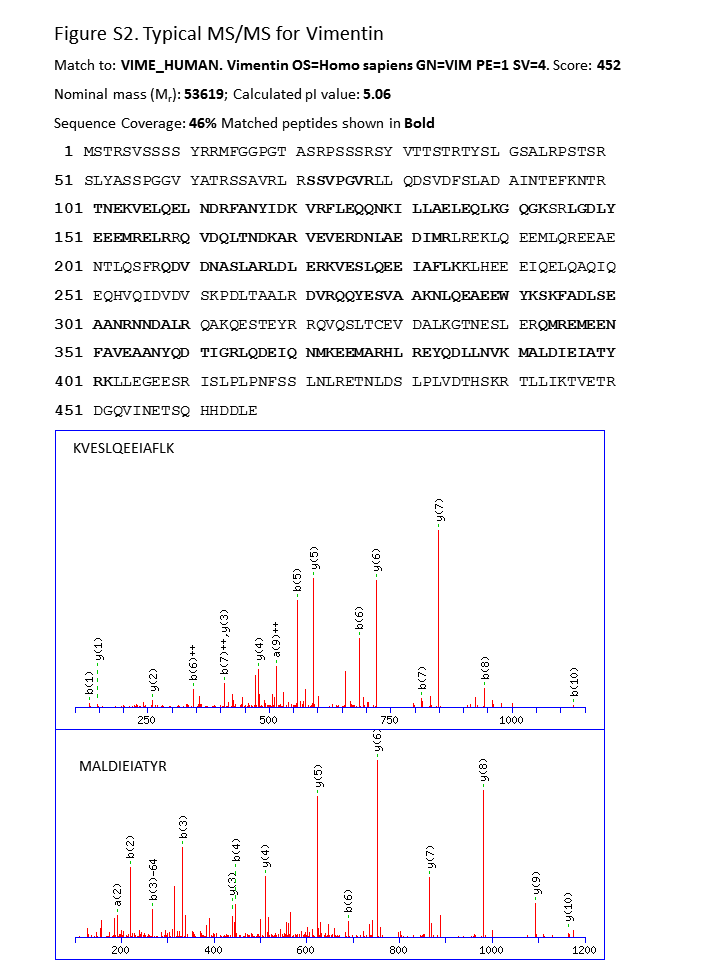

Supplement: Figure S2 — Typical MS/MS result of Vimentin in spots cut from 2D gel. Sequence coverage and matched peptides are shown in Bold. Two representativeMS/MS spectra of identified peptides were shown. (TIF) [file pone.0080779.s002.tif]

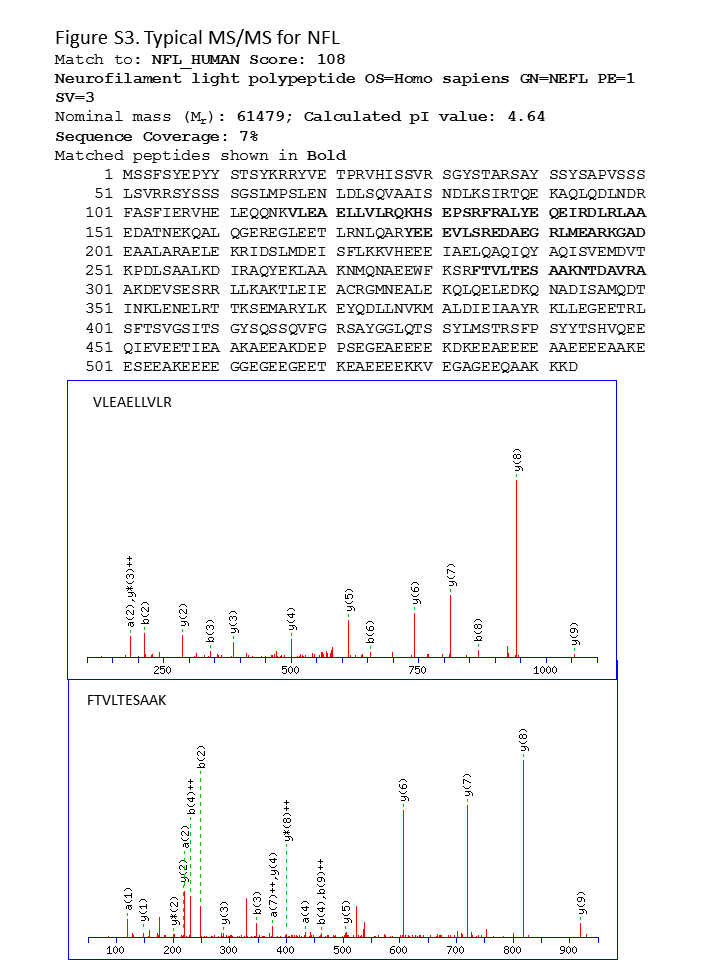

Supplement: Figure S3 — Typical MS/MS result of NFL in spots cut from 2D gel. Sequence coverage and matched peptides are shown in Bold. Two representativeMS/MS spectra of identified peptides were shown. (TIF) [file pone.0080779.s003.tif]

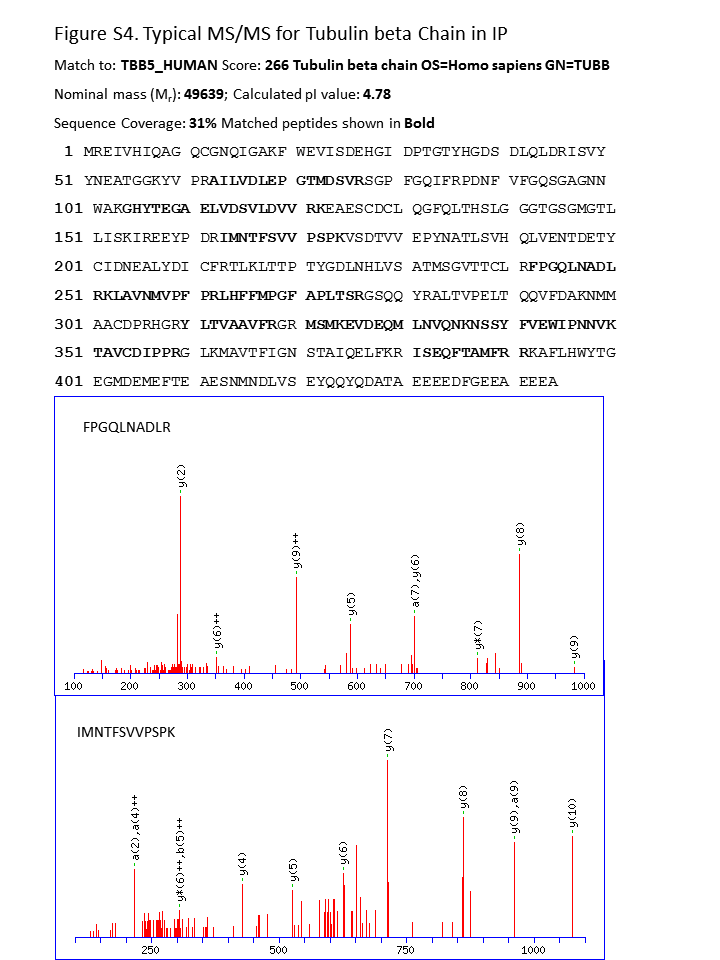

Supplement: Figure S4 — Typical MS/MS result of Tubulin beta Chain in bands cut from IP-SDS-PAGE gel. Sequence coverage and matched peptides are shown in Bold. Two representative MS/MS spectra of identified peptides were shown. (TIF) [file pone.0080779.s004.tif]

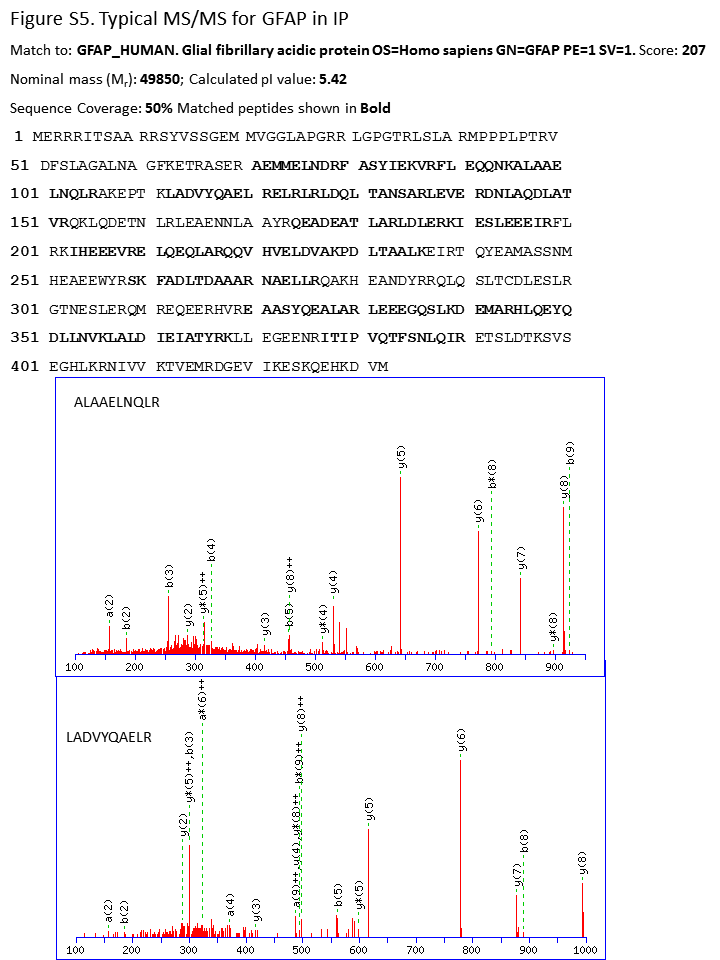

Supplement: Figure S5 — Typical MS/MS result of GFAP in bands cut from IP-SDS-PAGE gel. Sequence coverage and matched peptides are shown in Bold. Two representative MS/MS spectra of identified peptides were shown. (TIF) [file pone.0080779.s005.tif]

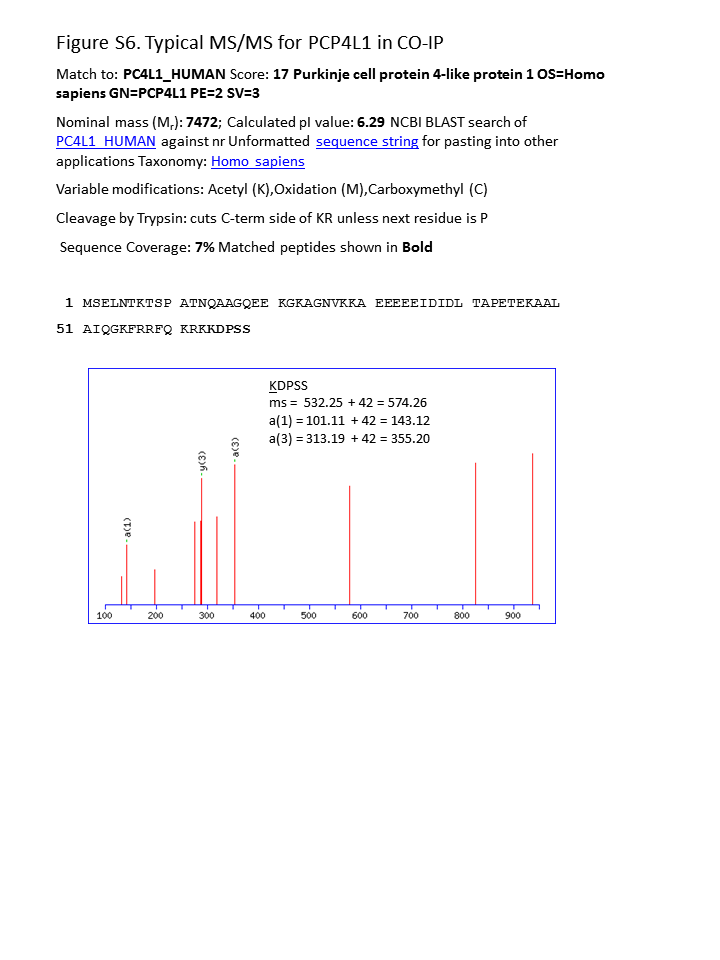

Supplement: Figure S6 — Typical MS/MS result of PCP4L1 in bands cut from IP-SDS-PAGE gel. Sequence coverage and matched peptides are shown in Bold. Two representative MS/MS spectra of identified peptides were shown. (TIF) [file pone.0080779.s006.tif]

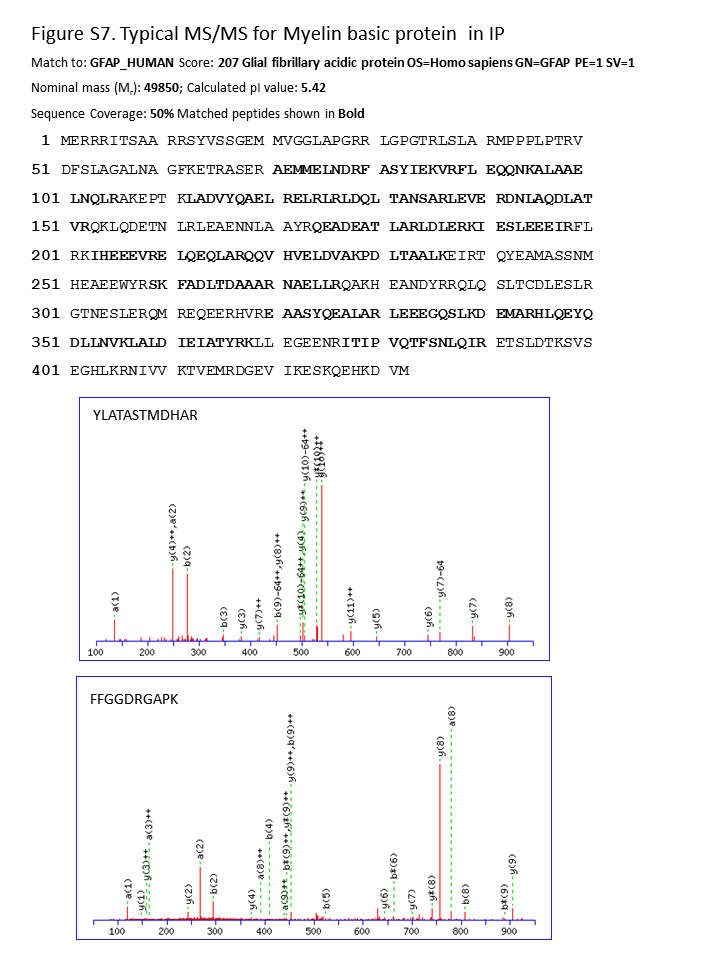

Supplement: Figure S7 — Typical MS/MS result of Myelin basic protein in bands cut from IP-SDS-PAGE gel. Sequence coverage and matched peptides are shown in Bold. Two representative MS/MS spectra of identified peptides were shown. (TIF) [file pone.0080779.s007.tif]

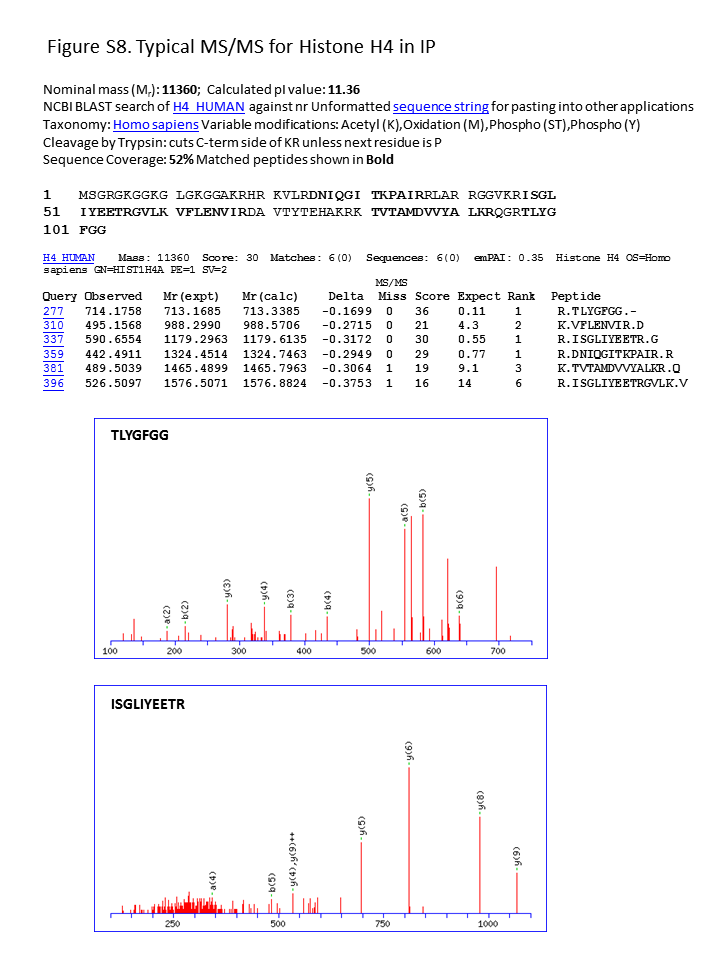

Supplement: Figure S8 — Typical MS/MS result of Histone H4 in bands cut from IP-SDS-PAGE gel. Sequence coverage and matched peptides are shown in Bold. Two representative MS/MS spectra of identified peptides were shown. (TIF) [file pone.0080779.s008.tif]
