# Supplementary material for: Proteomic Analysis Reveals Differentially Regulated Protein Acetylation in Human Amyotrophic Lateral Sclerosis Spinal Cord
Source: PLoS One. 2013 Dec 2;8(12):e80779. doi: 10.1371/journal.pone.0080779 (PMC3846615; doi:10.1371/journal.pone.0080779)
Supplement: Table S1 — Post-mortem spinal cord tissues used in the present study. Age-matched postmortem spinal cords were dissected within 48 hours. ∼5 mm3 of the same tissues were prepared as described in Materials and Methods. Aliquots were stored at −80°C until use. (DOC) [file pone.0080779.s009.doc]

**Table S1. Post-mortem spinal cord tissues used in the present study^a^**

|  | Subject # | Age | Sex | Type |
| --- | --- | --- | --- | --- |
| ALS | BB-1007 | 62 | Male | Thoracic |
|  | BB-1031 | 61 | Male | Thoracic |
|  | BB-1066 | 60 | Male | Thoracic |
|  | BB-1099 | 62 | Male | Thoracic |
| Average |  | 61±1 |  |  |
| Non-ALS | TB4 | 64 | Male | Thoracic |
|  | TB7 | 60 | Male | Thoracic |
|  | TB9 | 64 | Male | Thoracic |
|  | TB12 | 63 | Male | Thoracic |
| Average |  | 63±2 |  |  |

^a^ Age-matched postmortem spinal cords were dissected within 48 hours. ~5 mm^3^ of the same tissues were prepared as described in Materials and Methods. Aliquots were stored at -80 ˚C until use.
